# Supplementary figures and images for: Intrathecal morphine exacerbates paresis with increasing muscle tone of hindlimbs in rats with mild thoracic spinal cord injury but without damage of lumbar α-motoneurons
Source: PLoS One. 2022 Aug 15;17(8):e0273095. doi: 10.1371/journal.pone.0273095 (PMC9377624; doi:10.1371/journal.pone.0273095)

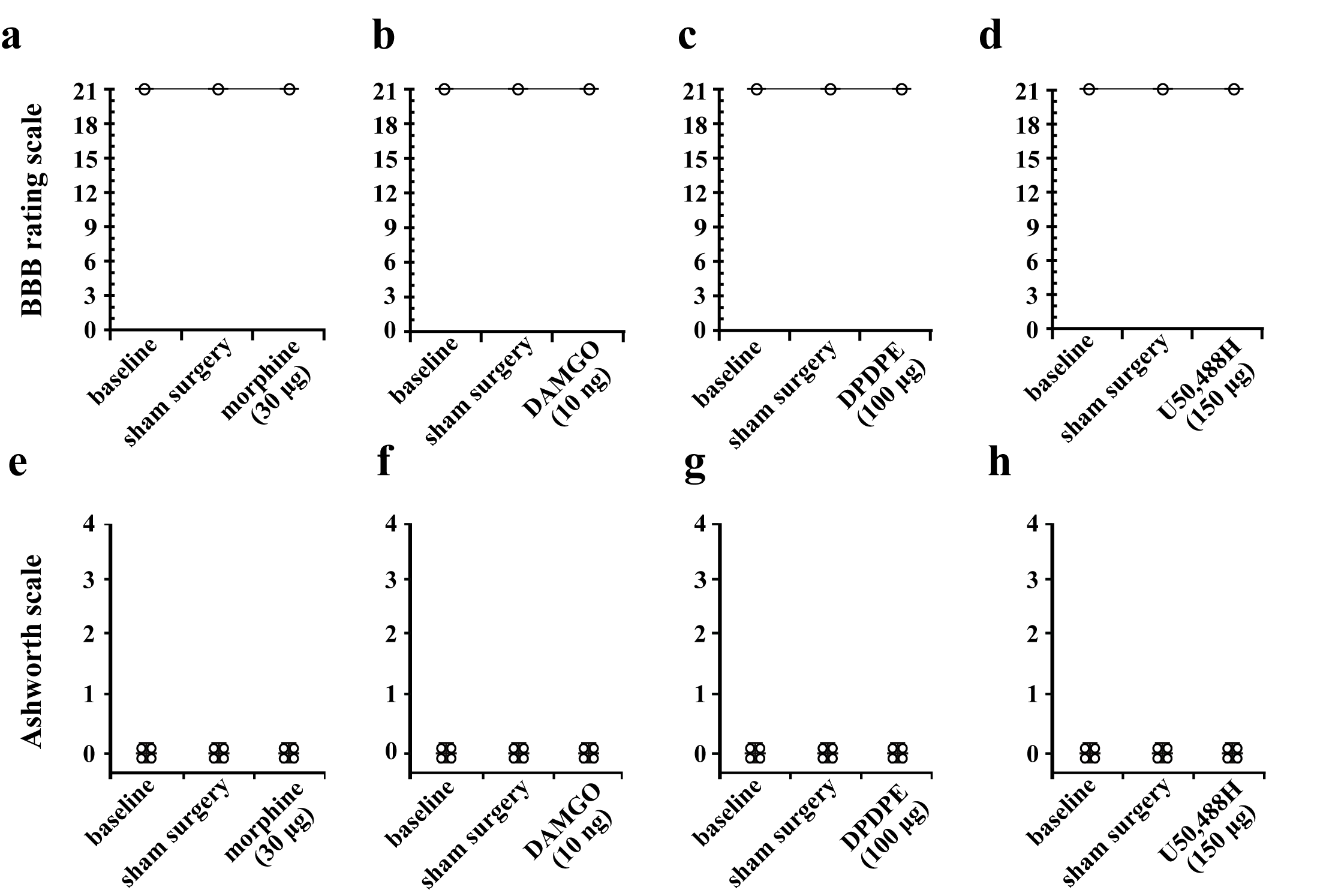

Supplement: S1 Fig — Locomotor function (a-d) and muscle tone (e-h) after intrathecal (IT) administration of morphine and μ- (DAMGO), δ- (DPDPE), and κ- (U50,488H) selective opioid receptor agonists after sham surgery without spinal cord injury. Locomotor function was evaluated using the 21-point Basso, Beattie, and Bresnahan (BBB) locomotor rating scale (0 = complete paralysis, 21 = normal locomotion) before sham surgery (baseline), 6 hours after sham surgery (sham surgery), and 30 min after administration of each drug. The panels show effects of (a) morphine, (b) DAMGO, (c) DPDPE, and (d) U50,488H. Data of the BBB score are presented as mean ± SDs. The Ashworth scales (0 = no increase in tone; 4 = increase in muscle tone) were used to evaluate changes in muscle tone of the hindlimbs before sham surgery (baseline), 6 hours after sham surgery (sham surgery), and 30 min after administration of each drug. The panels show effects of (e) morphine, (f) DAMGO, (g) DPDPE, and (h) U50,488H. Data are presented as scatter dot plots of the Ashworth score displaying the median as a line and the 25–75 percentiles. n = 4 for each dose. (TIF) [file pone.0273095.s002.tif]

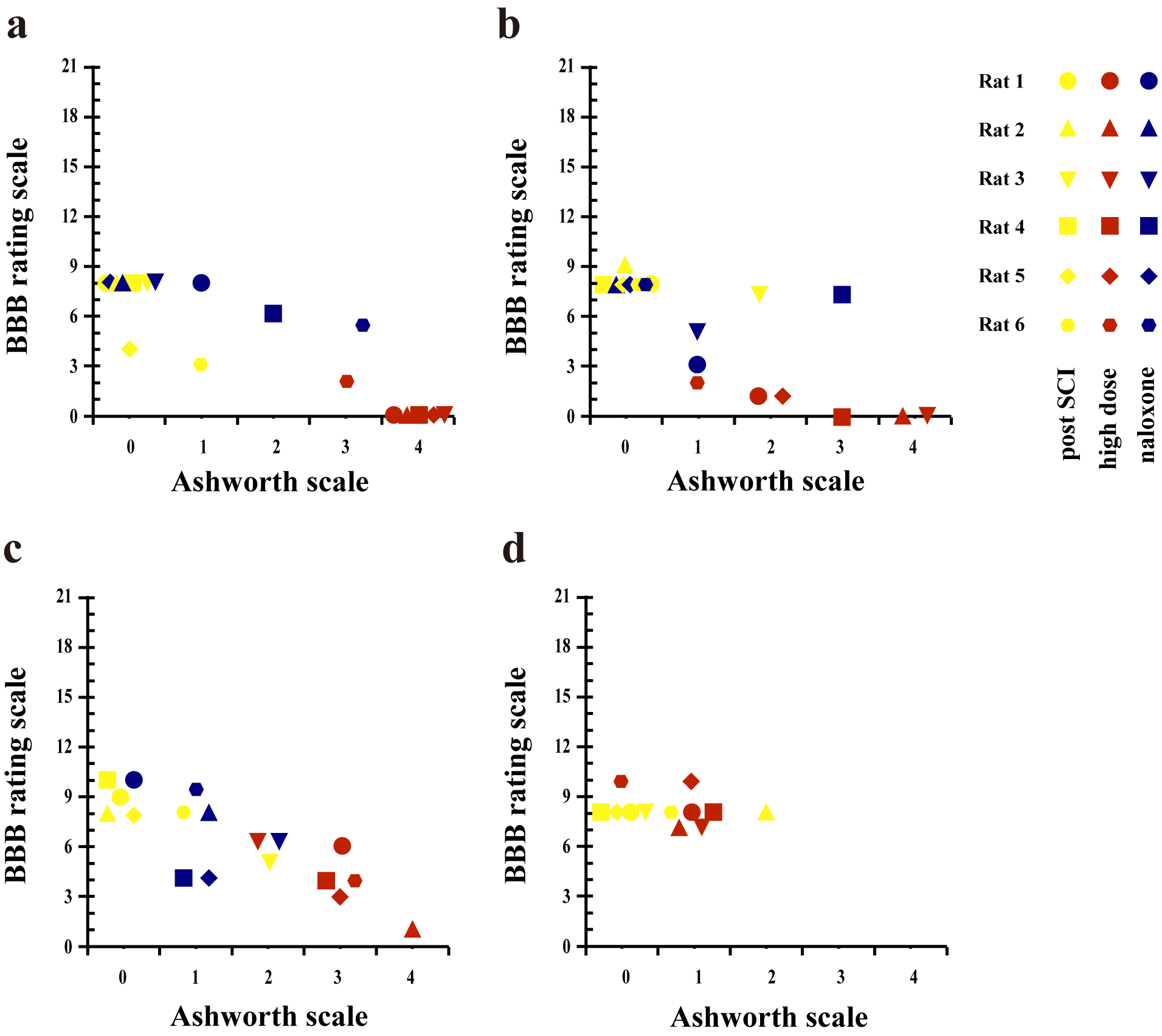

Supplement: S2 Fig — Scatter plots showing the relationship between the BBB score (y-axis) and the Ashworth score (x-axis) 6 hours after mild SCI (yellow), 30 min after intrathecal administration of 30 μg of morphine (panel a), 10 ng of DAMGO (μ-opioid receptor agonist, panel b), 100 μg of DPDPE (δ-opioid receptor agonist, panel c), and 150 μg of U50,488H (κ-opioid receptor agonist, panel d) (red) and 30 min after intrathecal administration of naloxone (opioid receptor antagonist) (blue). Naloxone was not used in the rats receiving U50,488H because the BBB scores did not decrease after administration of U50,488H. The plot shows the data for all rats (n = 6 in each group), with each rat indicated by a different shaped mark. (TIF) [file pone.0273095.s003.tif]
