# Supplementary material for: Intrathecal morphine exacerbates paresis with increasing muscle tone of hindlimbs in rats with mild thoracic spinal cord injury but without damage of lumbar α-motoneurons
Source: PLoS One. 2022 Aug 15;17(8):e0273095. doi: 10.1371/journal.pone.0273095 (PMC9377624; doi:10.1371/journal.pone.0273095)
Supplement: Attachment — Submitted filename: Responces to Reviewers.docx [file pone.0273095.s004.docx]

We would like to thank the academic editor and reviewer for carefully reading out manuscript (PONE-D-21-29555) and for the insightful comments. The comments led us to an improvement of the work. Detailed responses to the academic editor and reviewer are shown below. The comments of the academic editor and reviewer are reported in black and our reply is reported in red. In this revised version, changes to our manuscript within the document were all highlighted by using red-colored text (with underlining) in the file named “Revised Manuscript”.

**Reply to the Academic editor:**

PONE-D-21-29555

Intrathecal morphine exacerbates paresis with increasing muscle tone of hindlimbs in rats with mild thoracic spinal cord injury but without damage of lumbar a-motoneurons

PLOS ONE

Dear Dr. Tanaka,

Thank you for submitting your manuscript to PLOS ONE. After careful consideration, we feel that it has merit but does not fully meet PLOS ONE’s publication criteria as it currently stands. Therefore, we invite you to submit a revised version of the manuscript that addresses the points raised during the review process.

==============================
ACADEMIC EDITOR:

While the Reviewer found the work interesting, there are some issues that needs to be addressed.   Please review the comments and we encourage you to resubmit.
==============================

Please submit your revised manuscript by Jan 03 2022 11:59PM. If you will need more time than this to complete your revisions, please reply to this message or contact the journal office at [plosone@plos.org](mailto:plosone@plos.org). When you're ready to submit your revision, log on to [https://www.editorialmanager.com/pone/](https://www.editorialmanager.com/pone/) and select the 'Submissions Needing Revision' folder to locate your manuscript file.

Please include the following items when submitting your revised manuscript:

- A rebuttal letter that responds to each point raised by the academic editor and reviewer(s). You should upload this letter as a separate file labeled 'Response to Reviewers'.
- A marked-up copy of your manuscript that highlights changes made to the original version. You should upload this as a separate file labeled 'Revised Manuscript with Track Changes'.
- An unmarked version of your revised paper without tracked changes. You should upload this as a separate file labeled 'Manuscript'.

If you would like to make changes to your financial disclosure, please include your updated statement in your cover letter. Guidelines for resubmitting your figure files are available below the reviewer comments at the end of this letter.

If applicable, we recommend that you deposit your laboratory protocols in [protocols.io](http://protocols.io/) to enhance the reproducibility of your results. Protocols.io assigns your protocol its own identifier (DOI) so that it can be cited independently in the future. For instructions see: [https://journals.plos.org/plosone/s/submission-guidelines#loc-laboratory-protocols](https://journals.plos.org/plosone/s/submission-guidelines#loc-laboratory-protocols). Additionally, PLOS ONE offers an option for publishing peer-reviewed Lab Protocol articles, which describe protocols hosted on [protocols.io](http://protocols.io/). Read more information on sharing protocols at [https://plos.org/protocols?utm_medium=editorial-email&utm_source=authorletters&utm_campaign=protocols](https://plos.org/protocols?utm_medium=editorial-email&utm_source=authorletters&utm_campaign=protocols).

We look forward to receiving your revised manuscript.

Kind regards,

Warren Joseph Alilain, PhD
Academic Editor
PLOS ONE

Journal Requirements:

When submitting your revision, we need you to address these additional requirements.

1. Please ensure that your manuscript meets PLOS ONE's style requirements, including those for file naming. The PLOS ONE style templates can be found at 
[https://journals.plos.org/plosone/s/file?id=wjVg/PLOSOne_formatting_sample_main_body.pdf](https://journals.plos.org/plosone/s/file?id=wjVg/PLOSOne_formatting_sample_main_body.pdf) and 
[https://journals.plos.org/plosone/s/file?id=ba62/PLOSOne_formatting_sample_title_authors_affiliations.pdf](https://journals.plos.org/plosone/s/file?id=ba62/PLOSOne_formatting_sample_title_authors_affiliations.pdf)

We revised our manuscript to meet PLOS ONE’s style requirement.

2. Please include the following information relating to animal experiments in your Methods section:

-The frequency of animal monitoring, including the specific criteria you used to monitor animal health;
-Animal welfare considerations, including efforts to alleviate suffering, such as:
-Postoperative care,
-The method of euthanasia

Thank you for drawing this problem to our attention.

In the postoperative care, infection prevention and measures for dysuria were implemented. The use of antibiotics was described in line 103 in the original version. After the surgeries, general activity of rats was monitored every day. Each rat’s bladder was manually squeezed twice daily until voiding was re-established, then once a day for the duration of the study. Lactated ringer (5 ml) was injected subcutaneously once a day.

All surviving rats on day 14, as well as all animals reaching ethical endpoints during the experiment, were euthanized with sevoflurane excess inhalation. Ethical endpoints were defined as lethargy and self-mutilation of any body parts, and data from those animals (n = 5) were excluded from the study for analysis.

In addition, lidocaine (10 μg) (Sigma, St. Louis, MO, USA) was injected to confirm the location of the intrathecal catheter in this study. Rats that were not paralyzed by lidocaine were excluded from the study (n = 3).

In line 114, the following description (underlined) has been added to the “Methods” section in the revised manuscript.

After the surgeries, general activity of the rats was monitored every day. Each rat’s bladder was manually squeezed twice daily until voiding was re-established and then once a day for the duration of the study. Ringer’s solution (5 ml) was injected subcutaneously once a day. All surviving rats on day 14, as well as all animals reaching ethical endpoints during the experiment, were euthanized with sevoflurane excess inhalation. Ethical endpoints were defined as lethargy and self-mutilation of any body parts. During the experiment, five rats met the humane endpoints and were euthanized accordingly. Data from those animals were excluded from analysis.

In line 102, the following description (underlined) has been added to the “Methods” section in the revised manuscript.

Rats that were not paralyzed by lidocaine were excluded from the study (n = 3).

3. In your Data Availability statement, you have not specified where the minimal data set underlying the results described in your manuscript can be found. PLOS defines a study's minimal data set as the underlying data used to reach the conclusions drawn in the manuscript and any additional data required to replicate the reported study findings in their entirety. All PLOS journals require that the minimal data set be made fully available. For more information about our data policy, please see [http://journals.plos.org/plosone/s/data-availability](http://journals.plos.org/plosone/s/data-availability).

"Upon re-submitting your revised manuscript, please upload your study’s minimal underlying data set as either Supporting Information files or to a stable, public repository and include the relevant URLs, DOIs, or accession numbers within your revised cover letter. For a list of acceptable repositories, please see [http://journals.plos.org/plosone/s/data-availability#loc-recommended-repositories](http://journals.plos.org/plosone/s/data-availability#loc-recommended-repositories). Any potentially identifying patient information must be fully anonymized.

We prepared a supporting information file including all numerical data used for analysis and uploaded it on your editorial system.

In line 19, the following description (underlined) has been added to the “title page” in the revised manuscript.

Data Availability Statement: All relevant data are within the manuscript and its supporting information files.

Important: If there are ethical or legal restrictions to sharing your data publicly, please explain these restrictions in detail. Please see our guidelines for more information on what we consider unacceptable restrictions to publicly sharing data: [http://journals.plos.org/plosone/s/data-availability#loc-unacceptable-data-access-restrictions](http://journals.plos.org/plosone/s/data-availability#loc-unacceptable-data-access-restrictions). Note that it is not acceptable for the authors to be the sole named individuals responsible for ensuring data access.

We will update your Data Availability statement to reflect the information you provide in your cover letter

4. We note that you have included the phrase “data not shown” in your manuscript. Unfortunately, this does not meet our data sharing requirements. PLOS does not permit references to inaccessible data. We require that authors provide all relevant data within the paper, Supporting Information files, or in an acceptable, public repository. Please add a citation to support this phrase or upload the data that corresponds with these findings to a stable repository (such as Figshare or Dryad) and provide and URLs, DOIs, or accession numbers that may be used to access these data. Or, if the data are not a core part of the research being presented in your study, we ask that you remove the phrase that refers to these data.

In line 249, we have deleted “data not shown” and have changed the manuscript as follows in the revised version.

“Thirty μg of morphine, 10 ng of DAMGO, 100 μg of DPDPE, and 150 μg of U50,488H did not affect the BBB and Ashworth scores in sham-operated rats without SCI (~~data not shown~~ S1 Fig).”

In addition, we added the figure legend of S1 Fig as follows in line 441.

Figure legend of S1 Fig

Locomotor function (a-d) and muscle tone (e-h) after intrathecal (IT) administration of morphine and μ- (DAMGO), δ- (DPDPE), and κ- (U50,488H) selective opioid receptor agonists after sham surgery without spinal cord injury (SCI). Locomotor function was evaluated using the 21-point Basso, Beattie, and Bresnahan (BBB) locomotor rating scale (0 = complete paralysis, 21 = normal locomotion) before sham surgery (baseline), 6 hours after sham surgery (sham surgery), and after administration of each drug. The panels show effects of (a) morphine, (b) DAMGO, (c) DPDPE, and (d) U50,488H. Data of the BBB score are presented as means ± SDs. The Ashworth scales (0 = no increase in tone; 4 = increase in muscle tone) were used to evaluate changes in muscle tone of the hindlimbs before sham surgery (baseline), 6 hours after (sham surgery), and after administration of each drug. The panels show effects of (e) morphine, (f) DAMGO, (g) DPDPE, and (h) U50,488H. Data are presented as scatter dot plots of the Ashworth score displaying the median as a line and the 25–75 percentiles. n = 4 for each dose.

(TIF)

[Note: HTML markup is below. Please do not edit.]

Reviewers' comments:

Reviewer's Responses to Questions

**Reply to the Reviewer:**

**Comments to the Author**

1. Is the manuscript technically sound, and do the data support the conclusions?

The manuscript must describe a technically sound piece of scientific research with data that supports the conclusions. Experiments must have been conducted rigorously, with appropriate controls, replication, and sample sizes. The conclusions must be drawn appropriately based on the data presented.

Reviewer #1: Partly

2. Has the statistical analysis been performed appropriately and rigorously?

Reviewer #1: Yes

3. Have the authors made all data underlying the findings in their manuscript fully available?

The [PLOS Data policy](http://www.plosone.org/static/policies.action#sharing) requires authors to make all data underlying the findings described in their manuscript fully available without restriction, with rare exception (please refer to the Data Availability Statement in the manuscript PDF file). The data should be provided as part of the manuscript or its supporting information, or deposited to a public repository. For example, in addition to summary statistics, the data points behind means, medians and variance measures should be available. If there are restrictions on publicly sharing data—e.g. participant privacy or use of data from a third party—those must be specified.

Reviewer #1: No

According to the PLOS data policy, we have prepared the supporting information file (spreadsheet made by Excel) including all of the data for figures before statistical analysis.

In line 19, we have added the sentence “Data Availability: All relevant data are within the manuscript and it’s supporting Information files.”

In line 438, we have added the sentence “**Supporting information**

The supporting information file (xlsx) includes data before statistical analysis.

4. Is the manuscript presented in an intelligible fashion and written in standard English?

PLOS ONE does not copyedit accepted manuscripts, so the language in submitted articles must be clear, correct, and unambiguous. Any typographical or grammatical errors should be corrected at revision, so please note any specific errors here.

Reviewer #1: Yes

5. Review Comments to the Author

Please use the space provided to explain your answers to the questions above. You may also include additional comments for the author, including concerns about dual publication, research ethics, or publication ethics. (Please upload your review as an attachment if it exceeds 20,000 characters)

Reviewer #1: One of the underlying goals of this work is to determine how a single dose of opioids influences lower extremity function acutely following a high thoracic injury where the HL MNs are presumably intact. A second goal is to determine how continuous delivery of opioids influences locomotor recovery over a slightly longer period. The key outcomes are the BBB scale for hindlimb function during stepping and the Ashworth scale that looks at hindlimb rigidity.

Overall, the study addresses important issues. Some of the data/results are of interest, however there are some fairly serious experimental design issues that render the data hard to interpret for both major components of the study.

Major issues:
The authors indicate multiple times that previous literature shows intrathecally delivered morphine to increase spinal cord injury lesion size, specifically around the epicenter of the injury site. The authors speculate that this phenomenon may contribute to the locomotor deficits that are seen in the study, however, no analysis was performed on the injury epicenter, spared white matter, or the extent of damage after SCI. This renders the results very hard to interpret, in particular in light of the issues mentioned below.

The study design does not allow the interpretation that 72h infusion of morphine induces a lasting functional difference, just a delay in recovery. The text should clearly reflect this.

Thank you for making an important point. We agree with the reviewer’s suggestion.

The purpose of the present study was to investigate the effects of morphine on locomotor function in the early phase of mild thoracic SCI and involvement of activation of opioid receptors. In the original version of this manuscript, we speculated morphine-induced histological further aggravation of the injury epicenter as a potential mechanism for delayed functional recovery after continuous infusion of morphine. As the reviewer pointed out, the lesion epicenter in the thoracic spinal cord was not evaluated histologically. We have changed the manuscript as follows and shorten the discussion about the potential mechanisms of delayed functional recovery after continuous infusion of morphine.

We have added the following sentences (red, underlined) and deleted redundant description (black, strikethrough) in the Discussion section in line 404.

“At present, it is not clear why the aggravated locomotor function after continuous infusion of morphine did not recover immediately after discontinuing morphine administration. In our study, the number of α-motoneurons int the lumbar spinal cord was evaluated, but the lesion epicenter in the thoracic spinal cord was not histologically investigated. Mechanisms other than involvement of activation of spinal opioid receptors could not be revealed from the present study. Further work will be necessary to clarify the mechanisms of morphine-induced deterioration in locomotor recovery from mild SCI. ~~One possibility is that continuous infusion of morphine exacerbates the inflammatory response, leading to persistent decline in locomotor function [5-7]. IT morphine administered continuously is distributed from the lumbar to mid-thoracic spinal levels [29]. Further damage of descending spinal tracts in the epicenter may be involved in delayed functional recovery after SCI.~~ ~~Activation of κ2-opioid receptors [30] and downregulation of spinal glutamate transporters [31-33] have been proposed as mechanisms by which tissue damage is exacerbated at the epicenter. Another possibility is that chronic exposure to morphine produces maladaptive plasticity changes in the nervous systems [34, 35].~~ “

We have added the following sentences (red, underlined) and deleted redundant description (black, strikethrough) in the “Discussion” section in line 420.

Some limitations exist regarding the present study. First, while DPDPE and U50,488H used in this study agonize δ1- and κ1-opioid receptors, respectively [36], involvement of other subtypes such as δ2- and κ2-opioid receptors remains unknown from the present study. Second, ~~locomotor function after continuous infusion of morphine recovered to near the baseline slightly later than that after continuous infusion of saline in our study.~~ locomotor function recovery assessed by the BBB rating scale has been reported to be correlated with the spared tissues. However, rats can recover nearly normal locomotion even if some damage remains in the epicenter region [37]. Histological assessment in the lesion epicenter is required in a future study to investigate how or whether morphine affects the spinal cord after mild injury. ~~Therefore, we cannot exclude the possibility that continuous infusion of morphine produces permanent changes in the epicenter of the thoracic spinal cord. Further work will be necessary to clarify the impact of opioids on locomotor function after SCI.~~

The graphs in Figures 1, 3a and 4 represent the data as continuous without scaling the x-axis for time. This is misleading and the graphs need to be re-done to clearly show what is continuous and the temporal relationships in the data.

Thank you for making a good point. We made new Figures 1, 3, and 4 with the X axis as time. We think the new figures will help the readers to easily understand the changes in the BBB scores and Ashworth scores.

More specifically:
Results:
It is a shame that the experiment was not extended out to day 28, because it appears that the significant difference shown in Figure 4 would be gone by days 21 or 28. This suggests that whatever impact continuous morphine had on the circuitry is not going to induce a change in terminal function. The relationship between Ashworth and BBB data should be investigated using a scatterplot to look for correlations on an animal by animal basis.

Unfortunately, we cannot provide information about locomotor function after 14 days from the SCI. As the reviewer pointed out, the BBB score of rats receiving continuous infusion of morphine at 14 days after SCI was significantly lower than that of rats receiving saline, although it was on a recovery trend (Figure 3).

We have added the following sentences in the “Discussion” section in line 398.

The BBB score of rats receiving 72-hour continuous infusion of morphine at 14 days after SCI was significantly lower than that of rats receiving saline, although it was on a recovery trend (Fig 3a). Therefore, it is not clear from the present study whether continuous infusion of morphine simply delays functional recovery or leads to permanent decline in locomotor function.

According to the reviewer’s suggestion, we made a new figure (S2. Fig) showing the relationship between the BBB and Ashworth scores based on the data in figure 2. As shown in S2. Fig, decreases in the BBB score after administration of morphine, DAMGO, and DPDPE were associated with increase in the Ashworth score, suggesting that further aggravation of locomotor function by the drugs is due to excessive increase in muscle tone.

We have added the sentences as follows in the “Results” section in line 249.

The decreases in the BBB score after intrathecal administration of morphine, DAMGO, and DPDPE were clearly associated with increase in the Ashworth score. The recovery of the BBB score after intrathecal administration of naloxone was related to reduction of the Ashworth score (S2 Fig).

We have added S2 Figure and its figure legend in line 457.

**S2 Fig. Relationship between locomotor function and muscle tone.**

Scatter plots showing the relationship between the BBB score (y-axis) and the Ashworth score (x-axis) 6 hours after mild SCI (yellow), 30 min after intrathecal administration of 30 μg of morphine (panel a), 10 ng of DAMGO (μ-opioid receptor agonist, panel b), 100 μg of DPDPE (δ-opioid receptor agonist, panel c), and 150 μg of U50,488H (κ-opioid receptor agonist, panel d) (red) and 30 min after intrathecal administration of naloxone (opioid receptor antagonist) (blue). Naloxone was not used in the rats receiving U50,488H because the BBB scores did not decrease after administration of U50,488H. The plot shows the data for all rats (n = 6 in each group), with each rat indicated by a different shaped mark.

(TIF)

MN counts. Please described if and how you used stereological principles and prevented double-counting MNs given that the sections were 5µm in diameter and MNs may appear in several consecutive sections. Also, more information is necessary for the reader to understand the criteria you used to identify both uninjured and injured or damaged MNs. Did you only count MNs with visible nucleoli? Did damaged MNs also have to be greater than 25µm in diameter?

In our study, motoneurons in the ventral horn, cell diameter > 25 μm, were selected under the microscope based on morphological criteria. As the reviewer suggested, it is possible that motoneurons were overlooked in a thin slice preparation. To overcome that problem, 5-8 transverse sections of each spinal cord every 400 μm (double counting can be prevented) were examined and the average number of the motoneurons in the left ventral horn was calculated in each animal (please see the supporting information file). We think that evaluation of multiple sections alleviates shortcomings of evaluating the number of motoneurons with a thin slice.

Only motoneurons with visible nuclei were counted in this study. Therefore, we cannot exclude the possibility that motoneurons that were damaged to the extent that the nuclei were considerably injured were overlooked. However, based on our results obtained from multiple sections under the same conditions between the 3 groups (Figure 5), it is unlikely that the persistent decline in locomotor function after continuous infusion of morphine to rats with mild SCI is due to loss of α-motoneurons in the lumbar spinal cord.

We have added the following explanations (underlined parts) in the ”Methods” section in line 143.

For systematic analysis, 5-8 sections of each spinal cord, collected every 400 μm, were examined. An α-motoneuron with a visible nucleus was defined on the basis of a diameter larger than 25 μm and location in lamina IX

The images in Figure 5 are confusing and the location of the MNs in a is quite different from in b and c. Are you certain the orientation of the sections are similar?

We made new Figure 5a, 5b and 5c according to the reviewer’s suggestion.

How long did you wait after introducing the next drug or drug concentration before assessing the BBB and ashworth? This is an important detail.

In the experiment for which results are shown in Figure 2, the BBB scores and Ashworth scores were evaluated at 6 hours after mild SCI. Then the drug (morphine, DAMGO, DPDPE, or U50,488H) was administered. Thirty min later, the BBB scores and Ashworth scores were evaluated and naloxone was administered. In the experiment for which results are shown in Figure 2, data were obtained with a single dosing procedure (not cumulative dosing procedure). In other words, each animal received a single dose of each drug (morphine, DAMGO, DPDPE, or U50,488H). When the highest doses of the drugs (30 μg morphine, 10 ng DAMGO, and 100 μg DPDPE) used in this study decreased the BBB scores in rats with mild SCI, naloxone was intrathecally administered at 30 min after washing out the drugs.

In line 170, we have added the following sentences in the “Methods” section.

Morphine (3, 10, or 30 μg/10 μl), DAMGO (1, 3, or 10 ng/10 μl), DPDPE (10, 30, or 100 μg/10 μl), or U50,488H (15, 45, or 150 μg/10 μl) was administered through the IT catheter 6 hours after the SCI or after the sham operation without SCI, and the BBB and Ashworth scores were evaluated at 30 min after administration of each drug. Naloxone (60 μg/15 μl) (Sigma, St. Louis, MO, USA), an opioid receptor antagonist, was then intrathecally injected to the rats with decrease in the BBB scores at the highest doses of the drugs (30 μg morphine, 10 ng DAMGO, 100 μg DPDPE, and 150 μg U50,488H), and the BBB and Ashworth scores were recorded again at 30 min after administration of naloxone.

In line 179, we have changed the following sentences in the “Methods” section.

All catheterized rats were randomly divided into three groups as follows: 1) contused rats that received continuous IT morphine, 2) contused rats that received continuous IT physiological saline, and 3) sham-operated rats without SCI that received continuous IT morphine. In these 3 groups, six hours after the SCI or sham operation, a single dose of morphine (30 μg/10 μl) or physiological saline (10 μl) was administered through the IT catheter followed by continuous infusion of morphine (3 μg/μl/hr) or physiological saline (1 μl/hr), and the BBB and Ashworth scores were evaluated 30 min after the start of continuous infusion of the drugs. This continuous infusion dose of IT morphine was chosen according to a previous study in which that dose had a sufficient antinociceptive effect in tail-flick and colorectal distension tests [16]. Continuous infusion was performed for 72 hours using a subcutaneously implanted osmotic pump (Alzet micro-osmotic pump, model 1003D. DURECT Corporation, Cupertino, CA, USA).

Some rats in each group 3 days after SCI or sham surgery were used for histological analysis of α-motoneurons in the lumbar spinal cord. Responses to IT naloxone (60 μg) were also examined at 30 min after the end of 72-hour continuous IT administration of morphine to rats with SCI, ~~In a separate group,~~ ~~60 μg of IT naloxone was administered. to the contused rats~~ ~~that received continuous IT morphine for 72 hours~~. ~~In another separate group, a~~ A single dose of 30 μg of IT morphine was administered to rats with SCI at 30 min after the end of 72-hour continuous infusion of physiological saline ~~to the rats that received 72-hour continuous infusion of physiological saline after SCI~~. ~~At 30 min after morphine administration~~ After another 30 min, 60 μg of IT naloxone was administered. The BBB and Ashworth scores were evaluated at 30 min after administration of each drug.

For the data shown in Figure 4, did you “wash out” morphine before delivering naloxone, and for how long?

The drugs remaining in the catheter were washed out by 15 μl of saline every time because internal volume of 15 cm-long PE-10 catheter is approximately 10 μl. In Figure 4, morphine in the catheter was washed out with 15 μl of saline and 30 min later, naloxone was administered intrathecally.

In line 104, we have added the following sentence (underlined) in the “Methods” section.

The drugs remaining in the catheter were always washed out with 15 μl of saline.

Discussion:
On pages 18 and 19 (lines 318 to 322) you discuss damage to the MNs, but there are a couple issues of logic. First of all, the section shown in Figure 5a shouldn’t have any injured MNs because it is a control animal that received morphine only. Thus, the lack of increase following injury is in question. The second logic problem is the presumption that a mild contusion injury at T9-10 did not “extend to the lumbar spinal cord”. No doubt that the frank injury and cavitation did not, but even a mild injury disrupts descending and propriospinal input onto motoneurons and pre-motor neurons which could definitely influence their “health”. This should be re-stated and re-interpreted. This is particularly important because of the discussion on lines 335- about the descending input into the lumbar spinal cord.

What you pointed out is important.

As the reviewer pointed out, some motoneurons in the lumbar spinal cord of rats were affected to the same extents in the 3 groups (Sham+Morphine, SCI+Saline, SCI+Morphine) as shown in Figure 5e. A similar number of motoneurons in rats without SCI was damaged. Therefore, damage of some motoneurons in the lumbar spinal cord was probably due to causes other than mild thoracic SCI.

Catheters were placed intrathecally in all 3 groups (figure 5). Placement of an intrathecal catheter is a minimally invasive procedure. However, chronically implanted intrathecal catheters induce damage in the spinal cord in contact with the catheter (Yaksh TL et al, Anesthesiology 1986; 64:54-66) and in the fascicles in rats (Sakura S et al, Anesthesiology 1996;85:1184-9), although neurologic function assessed by the tail flick test was normal. In other words, intrathecal catheter placement can induce subclinical damage in the spinal cord.

Another possible explanation is that disruption of the descending pathway at the thoracic spinal cord induces morphological changes of the lumbar motoneurons. It has been reported that SCI at the T9/T10 level results in degeneration of motoneurons with dendritic withdrawal or atrophy in the lumbar spinal cord (Wang H et al, Experimental Neurology 2015;271:368-378; Wang Y et al, elife 2018 DOI: <https://doi.org/10.7554/eLife.39016.001>). However, this is less likely because some motoneurons in rats without SCI were affected to the same extent as shown in figure 5.

Taken together, the results indicate the possibility that damage in some motoneurons in the lumbar spinal cord was due to mechanical contact of the intrathecal catheter, although it cannot be concluded because of the lack of a control group without insertion of an intrathecal catheter.

Thus, we have added the following sentences to the Discussion section in line 380.

As shown in Figure 5, approximately 15 percent of the α-motoneurons in the lumbar spinal cord were darkly stained (damaged) in all three groups with or without SCI and with or without morphine administration. It has been shown that intrathecal catheter placement can induce subclinical damage in the spinal cord and fascicles in contact with the catheter [29, 30]. Intrathecal catheters were placed in rats in all three groups in the present study. Therefore, damage of some α-motoneurons observed in the three groups is more likely to be due to mechanical contact of the intrathecal catheter than the thoracic SCI or morphine. Implantation of the intrathecal catheter but not continuous infusion of IT morphine per se would cause some loss of motoneurons in the lumbar spinal cord of rats with thoracic SCI. ~~Continuous infusion of IT morphine did not increase damage of α-motoneurons in the lumbar spinal cord (Fig 5), indicating~~ Taken together, the results suggest that the morphine-induced persistent decline in locomotor function is not due to loss of α-motoneurons in the lumbar spinal cord.

We have added the following references and adjusted the serial number of other citations.

29. Yaksh TL, Noueihed RY, Durant PA. [Studies of the pharmacology and pathology of intrathecally administered 4-anilinopiperidine analogues and morphine in the rat and cat.](https://pubmed.ncbi.nlm.nih.gov/2867722/) Anesthesiology. 1986; 64: 54-66. doi: 10.1097/00000542-198601000-00009.PMID: 2867722.

[30. Sakura S, Hashimoto K, Bollen AW, Ciriales R, Drasner K. Intrathecal catheterization in the rat. Improved technique for morphologic analysis of drug-induced injury.](https://pubmed.ncbi.nlm.nih.gov/8916837/) Anesthesiology 1996; 85:1184-1189. doi: 10.1097/00000542-199611000-00028.PMID: 8916837

We have moved the following sentences from line 350 to line 376 to discuss in one paragraph the state of the motoneurons of the lumbar spinal cord.

A previous study showed little damage of motoneurons at a distance of 4 mm from the contusive epicenter [28]. Therefore, it is presumed that the effects of mild contusion injury at the T9-T10 spinal level did not extend to the lumbar spinal cord. This is also supported by our results showing that mild thoracic SCI did not increase the number of damaged α-motoneurons in the lumbar spinal cord compared to sham surgery without SCI.

Figures:
Figure 1. This figure is important and interesting, but shows each time point as having the same value over time. This is very misleading and mis-represents the data. I suggest showing this as two separate graphs, one to illustrate the Pre-SCI, Sci, 30min and day 1, and one to show the whole dataset (without the 30min), with the x-axis scaled to time. Or, keep the data all on one graph but scale the x-axis to time.

Thank you for making a good point. According to your second suggestion, we made a new Figure 1 with the X axis as time.

Same thing for Figure 3a. This is very misleading and in fact doesn’t even indicate the influence of the single dose of morphine given prior to the 30min assessment. This is absolutely critical and needs to be shown in some other way.

We made a new Figure 3 with the X axis as time. We measured the BBB and Ashworth scores at 6 hours after SCI, and then administration of morphine or saline (bolus plus continuous infusion) was started. The BBB and Ashworth scores 30 min after the start of administration are shown at 6.5 hours in new Figure 3. We believe that the new figure makes it easy to understand the time relationship between morphine administration and locomotor function.

In Figure 2, although the and κ-opioid receptor agonist did not cause significant changes in function, Panels d and h should include the effects of reversing the κ-opioid receptor agonist with naloxone as Panels a-c/e-j showed for the other agonists and morphine.

In the present study, naloxone was not given after intrathecal administration of a κ-opioid receptor agonist (U50,488H) to rats with SCI because U50,488H (15 μg, 45 μg, 150 μg) used this study did not affect the BBB and Ashworth scores after mild SCI. The IT analgesic dose of U50,488H for antinociception and antiallodynic action on neuropathic pain is 25 μg [18, 19]. In other words, U50,488H at a dose that greatly exceeds the amount of analgesia did not affect the BBB and Ashworth scores, suggesting that activation of the κ-opioid receptor has little involvement in the morphine-induced aggravation of locomotor function after mild SCI. We performed this experiment to identify which opioid receptor types are involved in morphine-induced acute aggravation of locomotor function of animals with SCI. As the reviewer suggested, our results are not sufficient for a conclusion that activation of the κ-opioid receptor is not involved in morphine-induced aggravation of locomotor function at all. However, our results were sufficient to suggest that activation of μ- or δ-opioid receptors causes an increase in muscle tone, resulting in further aggravation of locomotor function.

We have added the following description (underlined parts) in the “Results” section in line 240.

In contrast, none of the doses of U50,488H tested in this study affected locomotor function after SCI, and therefore, naloxone was not used in this group.

In Figure 4a, the naloxone data is not collected on day 4 and thus the data points should not be shown connected. Same thing for figure 4b (morphine and naloxone).

　We are sorry that our figures were hard to understand.

In the experiment for which results are shown in Figure 4a, naloxone was intrathecally administered 30 min after the end of continuous infusion of morphine on day 3 and the BBB score was evaluated at 30 min after naloxone administration.

In the experiment for which results are shown in Figure 4b, morphine was intrathecally administered 30 min after the end of continuous infusion of saline on day 3 and the BBB score was evaluated at 30 min after morphine administration. Naloxone was administered 30 min later and the BBB score was evaluated at 30 min after naloxone administration.

We made new Figures 4a and 4b for clarifying the timing of drug administration in the revised manuscript.

In addition, we have added the following description (underlined parts) in the legend of Figure 4 in line 300.

**Fig 4. Effects of intrathecal (IT) naloxone, an opioid receptor antagonist, on locomotor function after administration of IT morphine to rats with mild spinal cord injury (SCI).** Locomotor function was evaluated using the 21-point Basso, Beattie, and Bresnahan (BBB) locomotor rating scale. Panel (a) shows effects of IT naloxone (60 μg), administered 30 min after the end of 72-hour continuous infusion of morphine, on locomotor function of rats with SCI ~~after continuous infusion of IT morphine~~. A single dose of 30 μg of IT morphine was administered followed by continuous infusion of 3 μg/hour morphine for 72 hours to rats with SCI. Panel (b) shows effects of IT naloxone on locomotor function after IT administration of a single dose of morphine. A single dose of 30 μg of IT morphine was administered 30 min after the end of 72-hour continuous infusion of normal saline to rats with SCI. After another 30 min, naloxone was intrathecally administered. The BBB scores on the third day were evaluated 30 min after administration of each drug. Data are shown as means ± SDs. n=6 in each group. *P < 0.05 compared to data immediately after thoracic SCI. # P < 0.05 compared to before IT naloxone.

To make it easier for the reader to understand the manuscript, we have changed or added the following sentences. (Add, ~~Remove~~)

In line 28, from “~~T9-10 spine level~~” to “T9-T10 spine levels”

In line 32, from “~~damaged and total~~” to “total and damaged”

In line 34, from “~~morphine aggravated~~” to “morphine temporarily aggravated”

In line 63, from “~~T12-13 spine level~~” to “T12-T13 spine levels”

In line 108 and line 114, from “a T9-10 laminectomy” to “a T9-T10 laminectomy”

In line 154,

A single dose of 30 μg of morphine (morphine hydrochloride; Daiichi Sankyo, Tokyo, Japan) in 10 μl of physiological saline (morphine group), ~~an analgesic dose to heat and electrical shock in rats with moderate thoracic SCI [6],~~ or 10 μl of physiological saline (saline group) was administered through the IT catheter 6 hours after SCI. Thirty μg of morphine was determined according to a previously described dose that has a sufficient antinociceptive effect to nociceptive stimuli by heat and electrical shock in rats with moderate thoracic SCI [6].

In line 182,

In these 3 groups, six hours after the SCI or sham operation, a single dose of morphine (30 μg/10 μl) or physiological saline (10 μl) was administered through the IT catheter followed by continuous infusion of morphine (3 μg/μl/hr) or physiological saline (1 μl/hr), and thereafter the BBB and Ashworth scores were evaluated 30 min after the start of continuous infusion of the drugs. This continuous infusion dose of IT morphine ~~has been shown to have an~~ was chosen according to a previous study in which that dose had a sufficient antinociceptive effect in tail-flick and colorectal distension tests [16].

In line 219, from “at the T9-10” to “at the T9-T10

In line 344,

Taken together, these results suggest that morphine-induced aggravation of locomotor function after thoracic SCI is, at least in part, attributed to hypertonia of the hindlimb muscles via activation of μ- and/or δ- opioid receptors but not κ- opioid receptors. Our results also suggest that the mechanism of morphine-induced aggravation of locomotor function varies depending on the duration of morphine administration in the early phase of SCI.

6. PLOS authors have the option to publish the peer review history of their article ([what does this mean?](https://journals.plos.org/plosone/s/editorial-and-peer-review-process#loc-peer-review-history)). If published, this will include your full peer review and any attached files.


If you choose “no”, your identity will remain anonymous but your review may still be made public.


**Do you want your identity to be public for this peer review?** For information about this choice, including consent withdrawal, please see our [Privacy Policy](https://www.plos.org/privacy-policy).

Reviewer #1: No

[NOTE: If reviewer comments were submitted as an attachment file, they will be attached to this email and accessible via the submission site. Please log into your account, locate the manuscript record, and check for the action link "View Attachments". If this link does not appear, there are no attachment files.]

While revising your submission, please upload your figure files to the Preflight Analysis and Conversion Engine (PACE) digital diagnostic tool, [https://pacev2.apexcovantage.com/](https://pacev2.apexcovantage.com/). PACE helps ensure that figures meet PLOS requirements. To use PACE, you must first register as a user. Registration is free. Then, login and navigate to the UPLOAD tab, where you will find detailed instructions on how to use the tool. If you encounter any issues or have any questions when using PACE, please email PLOS at [figures@plos.org](mailto:figures@plos.org). Please note that Supporting Information files do not need this step.

--------------------------------------------------------------------------------------------------------------

Again, thank you for giving us the opportunity to strengthen our manuscript with your valuable comments and queries.
