# Supplementary material for: Intrathecal morphine exacerbates paresis with increasing muscle tone of hindlimbs in rats with mild thoracic spinal cord injury but without damage of lumbar α-motoneurons
Source: PLoS One. 2022 Aug 15;17(8):e0273095. doi: 10.1371/journal.pone.0273095 (PMC9377624; doi:10.1371/journal.pone.0273095)
Supplement: Attachment — Submitted filename: Responses to Reviewers.docx [file pone.0273095.s006.docx]

We would like to thank the academic editor and reviewers for carefully reading our manuscript and for the insightful comments. The comments led to an improvement of the work. Detailed responses to the reviewers are shown below. The comments from the reviewers are shown in black and our replies are shown in red. In this revised version, changes to our manuscript within the document were all highlighted by using red-colored text (**with underlining**) in the file named “**Revised Manuscript with Track Changes**”.

**Reply to Reviewer #2**

**Reviewer #2:** The authors addressed all of my concerns. However, some of the amendments in the paper are difficult to follow. i.e., pp. 24, line 399-pp 25, line 412. The ideas seem a little muddled in this section. Please work to improve the clarity of this text, and the 2 separate points you are making:

We wish to thank the reviewer for making important suggestions. We have deleted the sentences in lines 397-412 of the revised manuscript with track changes of the previous version. We have added new sentences as described later.

**Reviewer #2:**　the limitation of the current study not using continuous naloxone together with continuous morphine,

The half-life of naloxone is approximately 10 min (Heyman JS et al., Life Sci 1986). The half-life of intrathecal morphine in humans is approximately 90 min ([Sjöström](https://pubmed.ncbi.nlm.nih.gov/?term=Sj%C3%B6str%C3%B6m+S&cauthor_id=2891329) S et al, Anesthesiology 1987). Therefore, investigation of the effects of continuous infusion of intrathecal naloxone during continuous infusion of morphine would be required in order to clarify the roles of opioid receptors in the morphine-induced persistent decline of locomotor function. However, it was not tested in our study. In addition, we investigated short-term effects of opioid receptor subtype-selective agonists on locomotor function (Fig. 2), but we did not investigate their long-term effects. Thus, our results did not reveal how activation of opioid receptors is involved in the persistent decline and delayed recovery of locomotor function in rats with mild SCI receiving continuous infusion of morphine for 72 hours.

In line 397, we have added the following sentences in the Discussion section.

These results suggest that the mechanisms of persistent decline in locomotor function caused by continuous infusion of morphine are different from those of acute and transient decline of locomotor function caused by a single dose of morphine. We investigated the short-term effects of opioid receptor subtype-selective agonists on locomotor function (Fig 2), but we did not investigate their long-term effects. Continuous infusion of naloxone would be required to completely block the activation of opioid receptors during continuous infusion of morphine because the half-life of naloxone is approximately 10 min [32]. However, it was not tested in this study. Therefore, our results did not reveal in detail how activation of opioid receptors is involved in the persistent decline and delayed recovery of locomotor function in rats with mild SCI receiving continuous infusion of morphine for 72 hours.

**Reviewer #2:**　the discrepancy between the current results and previous studies with kappa opioid receptor agonists.

In our study, a single dose of 0.08 μmol (30 μg) of morphine hydrochloride caused a transient deterioration of locomotor function after mild SCI via activation of μ- and δ-opioid receptors but did not affect functional recovery at 14 days after mild SCI (Fig 1). In addition, we observed that continuous infusion of morphine (0.67 μmol of morphine hydrochloride administered over a period of 3 days) caused acute deterioration and persistent decline of locomotor function after SCI (Fig 3).

It has been shown that a κ-opioid receptor antagonist (WIN44,441-3) promotes functional recovery from traumatic spinal cord injury in cats (Faden AI et al., Peptides, 1985). Aceves et al. (J Neurotrauma 2017) reported that pretreatment with nor-Binaltorphimine (norBNI), a selective κ-opioid receptor antagonist, blocked the adverse effects of 0.32 μmol of morphine sulfate on long-term recovery of locomotor function at 2 to 3 weeks after SCI. In addition, it has been shown that administration of GR89696, a selective κ-opioid receptor agonist, does not cause acute deterioration of locomotor function but undermines recovery after SCI (Aceves M et al., Spinal Cord 2016). These results indicate that the κ-opioid receptor plays a critical role in the morphine-induced attenuation of locomotor recovery. It has been suggested that a κ-opioid receptor antagonist may prevent the morphine-induced persistent decline of locomotor function by reducing the extent of cell death at the site of injury [Aceves 2017].

It should be noted that morphine did not cause acute deterioration of locomotor function but delayed functional recovery in the SCI model of Aceves et al. Locomotor function at 1 day after moderate SCI in their study was lower than that in our study using rats with mild SCI. Therefore, it is likely that there was no room for further acute decline of locomotor function due to morphine administration in their model.

In summary, rats with mild SCI in which locomotor function was preserved to some extent were used in our study unlike in previous studies. Our results suggest that activation of μ- and δ-opioid receptors is involved in the morphine-induced acute deterioration of locomotor function in animals with mild SCI, but the role of opioid receptors in morphine-induced delayed functional recovery was not examined. In contrast, previous studies have demonstrated significant roles of the κ-opioid receptor in long-term recovery of locomotor function in animals with moderate SCI receiving morphine. The novelty of our study is that we showed that morphine can cause acute deterioration of residual locomotor function in animals with mild SCI via activation of opioid receptors. The results of our study taken together with the results of previous studies [6, Aceves 2016, 2017] suggest that different subtypes of opioid receptors are involved in morphine-induced acute deterioration of locomotor function and delayed recovery.

In order to make it easier to understand the differences between the previous studies and the present study, we have added the following sentences in the Discussion section in line 412.

Antagonism of the κ-opioid receptor has been reported to attenuate the morphine-induced persistent decline of locomotor function by reducing the extent of cell death at the site of injury [33]. It has also been shown that an agonist of the κ-opioid receptor undermines the recovery of locomotor function after a moderate degree of SCI [34]. These results indicate that the κ-opioid receptor plays a critical role in the morphine-induced attenuation of locomotor recovery. It should be noted that there was a difference in the degrees of SCI in those previous studies [33, 34] and our study. Locomotor function at 1 day after SCI in the previous studies was lower than that in our study using rats with mild SCI. Morphine-induced acute deterioration of locomotor function after SCI, which was observed in our study, may occur only in the case of mild SCI in which locomotor function is preserved to some extent. The results of our study taken together with the results of those previous studies [33, 34] suggest that morphine has various impacts on residual locomotor function after mild SCI, including acute deterioration and attenuation of recovery, via different subtypes of opioid receptors.

**Reviewer #2:** Also consider rephrasing from "time to time."

Thank you for pointing out the confusing expression.

Naloxone could reverse morphine-induced acute deterioration of locomotor function at 30 min after morphine administration (Figs. 2a and 4b). In contrast, a single dose of naloxone could not reverse the morphine-induced persistent decline of locomotor function at 72 hours after the start of continuous infusion (Fig. 4a). The role of opioid receptors may vary with the time elapsed after morphine administration. Therefore, we used the expression “time to time”.

Nonetheless, as the reviewer pointed out, "time to time" is vague and unclear, so we have deleted it.

**Reviewer #2:** I assume you mean with acute activation of the opioid receptors versus more chronic engagement of the opioid receptors with continual morphine?

The half-life of naloxone is approximately 10 min (Heyman JS et al., Life Sci 1986). The half-life of intrathecal morphine in humans is approximately 90 min ([Sjöström](https://pubmed.ncbi.nlm.nih.gov/?term=Sj%C3%B6str%C3%B6m+S&cauthor_id=2891329) S et al., Anesthesiology 1987). The duration of analgesia with morphine is short. Therefore, repeated or continuous administration is necessary for long-term analgesic effects. It is unlikely that a single administration of morphine causes chronic activation of opioid receptors.

In our study, a single dose of morphine was given to produce transient activation of opioid receptors, and continuous infusion of morphine for 72 hours was given to produce prolonged activation of opioid receptors.

There was a typo. We corrected it as follows.

In line 453, “U50,488H418" to “U50,488H''

We have added the following reference and adjusted the serial number of other citations.

34. Aceves M, Mathai BB, Hook MA. Evaluation of the effects of specific opioid receptor agonists in a rodent model of spinal cord injury. Spinal Cord. 2016 ;54(10): 767-777. https://doi.org/10.1038/sc.2016.28 PMID: 26927293

Again, thank you for giving us the opportunity to strengthen our manuscript with your valuable comments and queries.
